# Et tu Brute? The Roles of Subordinate–Supervisor Similarities on the Relationship between Abusive Supervision and Employee Silence Behavior: A Study from the Subordinates’ Perspectives

**DOI:** 10.3390/bs14070582

**Published:** 2024-07-09

**Authors:** Pınar Bayhan Karapinar, Ozge Tayfur Ekmekci, Selin Metin Camgoz, Sergio López Bohle, Eren Miski Aydin

**Affiliations:** 1 Department of Business Administration, Faculty of Economics and Administrative Sciences, Beytepe Campus, Hacettepe Üniversitesi, 06800 Ankara, Turkey; otayfur@hacettepe.edu.tr (O.T.E.); selinm@hacettepe.edu.tr (S.M.C.); erenma@hacettepe.edu.tr (E.M.A.); 2 Departamento de Administración, Facultad de Administración y Economía, Universidad de Santiago de Chile, Santiago 9170022, Chile; sergio.lopez@usach.cl

**Keywords:** abusive supervision, employee silence behavior, gender similarity, perceived subordinate–supervisor similarity, relational demographic approach

## Abstract

The current study examined the moderating effects of subordinate–supervisor similarities on abusive supervision and employee silence relationships. We addressed the question of whether employees’ silence reactions are alleviated or aggravated when the abuse comes from a supervisor who shares a similar gender and other sociodemographic attributes with the employee. The results indicated that abusive supervision led to more silence behavior and supported the moderating effect of perceived sociodemographic similarity on this relationship. However, regardless of gender similarities with their supervisors, the findings postulated that employees experiencing abusive supervision were more likely to remain silent at work. When there is a perceived sociodemographic similarity between the employee and the supervisor, abusive supervision has been found to have a harsher influence on employee’s silence behavior. These findings help us better understand the antecedents of employee silence behavior and provide important implications for subordinate–supervisor similarity dynamics in exposure to abusive supervision.

## 1. Introduction

Employee silence, referring to the intentional withholding of information, opinions, suggestions, or concerns about potentially important organizational issues [1,2], has received considerable attention in organizational studies [1,3]. Researchers have linked it to decreased job satisfaction [4], poor task performance, fewer organizational citizenship behaviors [5], and increased deviant behavior [6], all of which are highly dysfunctional in the workplace.

While the previous literature mostly concentrates on the consequences of employee silence [7], there has been little research on the mechanisms behind employees’ silence, with less focus on identifying the factors that prevent employees from being silent. A recent meta-analysis [7] indicated several predictors of employee silence such as individual dispositions (e.g., higher levels of agreeableness and neuroticism, lower levels of conscientiousness and extraversion, proactive personality, and higher levels of negative affect) and job perceptions and beliefs (e.g., lower levels of psychological safety, organizational identification, organizational justice, psychological contract, social support, higher levels of organizational cynicism, and job autonomy) [7]. The same meta-analysis also emphasized leader-related factors (e.g., abusive supervision, authoritarian leadership, LMX, and trust in the leader) as important antecedents of employees’ silence behaviors [1,7]. Thus, this paper initially expands the previous research by exploring the influence of abusive supervision on employee silence based on the Conservation of Resource Theory (COR) [8], a widely recognized stress reaction theory.

Since abusive supervision is interpersonal behavior between superiors and subordinates [9] and depends on subordinates’ perceptions [10], not all subordinates can react similarly. Therefore, different moderating factors could potentially influence the association between abusive leadership and silence behavior. This paper therefore examines how and under what conditions abusive supervision could relate to alleviating or aggravating the employees’ behavior of intentionally withholding opinions. At this point, we address whether the perceived similarities/ dissimilarities in demographic attributes of subordinates and supervisors could moderate the influence of abusive supervision on employee silence behavior. More precisely, we posed the crucial question of how subordinates react to mistreatment from superiors that they believe to be similar. We propose and test that shared attributes can influence the dynamics of the relationship and the employee’s perception and reaction to the abuse. It is important to note that the focus of this study is exclusively on the followers’ perceptions of their supervisors’ similarities.

Positioning gender as an important (dis)similarity factor, prior studies display that individuals with different genders are inclined to have different perspectives on the same events, and further argue that gender dissimilarity between subordinate and supervisor could result in reduced communication among parties [11]. Beyond gender effects, some studies have examined sociodemographic characteristics including age, race, educational background, tenure, and ethnicity regarding superior–subordinate similarity factors [12,13,14,15]. Those studies suggest that increased dissimilarity in subordinate–supervisor demographic characteristics is associated with less desirable employee and organizational outcomes [15]. However, the evidence for dis/similarities in employee outcomes is (still) not fully understood [16], and more research is necessary to further in-depth comprehension of this phenomenon [17].

**Figure 1:**
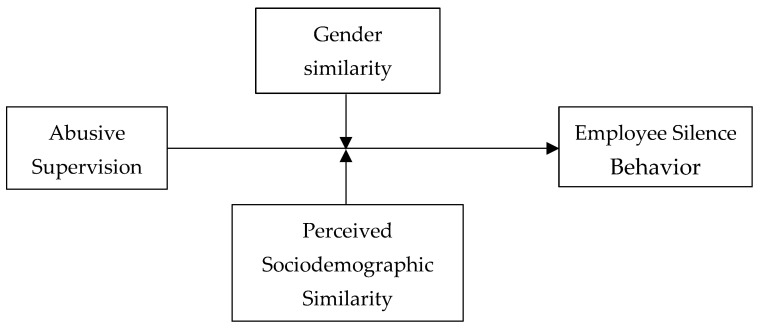
The proposed model.

Drawing from a social identity theory [18,19], we propose a theoretical framework highlighting the direct effect of abusive supervision and the moderating effect of perceived (dis)similarities in a given demographic attribute of subordinates and superiors to predict employee silence. To this end, this paper explores how a specific leadership style of abusive supervision may interact with perceived (dis)similarities between superior and subordinate and jointly influence employees’ silence behavior. In particular, we argue that the magnitude of the relationship between abusive supervision and employee silence behavior could vary based on gender and perceived sociodemographic similarities between superiors and subordinates. Addressing those similarities as important resources [20], we question whether this resource buffers or mitigates the negative impact of abusive supervision on silence behavior. Figure 1 depicts our proposed study model. 

This study makes several significant contributions to the literature. First, scholars [15] highlight that individuals’ demographic characteristics do not solely depend on one demographic attribute but vary on multiple attributes, thus one needs to explore the full impacts of the demographic characteristics on relevant outcomes. In this regard, the current study extends the literature by exploring the effects of gender and perceived sociodemographic similarities including age, educational background, ethnicity, social status, and cultural similarities simultaneously in predicting employee silence. Similarly, only a few studies have treated (dis)similarity issues as moderating factors [17,21]. Therefore, we also contribute to how perceived (dis) similarities between subordinates and superiors as moderating factors could influence the association between abusive supervision and the silence behaviors of the followers. Third, while much is now known about the impact of constructive leadership styles [17] and their interaction with gender similarity on employee outcomes, we focus on abusive supervision as a form of destructive leadership. As there has been no previous work on the joint effect of abusive leadership and subordinate–supervisor dis/similarity in employee silence, we address this gap in understanding the phenomena. 

The fourth contribution of this study is its focus on a non-Western sample derived from Türkiye. Turkish culture is often characterized as a blend of ‘Eastern’ and ‘Western’ values [22]. Turkish culture has been classified as being relatively high in collectivistic and power distant characteristics [23]. Despite cultural shifts in Turkish households, where autonomy and self-determination are now considered important cultural values [24], collectivism and power distance remain deeply ingrained in Turkish organizations and society [22]. Members of collectivistic cultures may therefore feel more exposed to the distinctiveness threat than members of individualistic cultures that place greater emphasis on individuality and independence [23,25]. This distinctiveness threat could shape the dynamics of leader–follower relationships by shaping the importance attached to perceived similarities/dissimilarities between subordinates and supervisors. As such, individuals from collectivistic societies may feel more exposed to the threat of distinctiveness than those from individualistic cultures that value individuality and independence [26]. Moreover, the members of collectivist cultures tend to value harmonious relationships with others such as supervisors and coworkers, therefore they would be more sensitive when such harmonious relationships were threatened. 

Moreover, power distance, which involves “... the extent to which the less powerful members of organizations and institutions accept and expect that power is distributed unequally” [23], may also shape leader–follower relationships. Members of highly power distant cultures accept the inequalities in the distribution of power and need no justification for such inequalities.

Therefore, relatively high power distance could influence the plausible impact of mistreatment from supervisors.

## 2. The Relationship between Abusive Supervision and Employee Silence

Abusive supervision refers to “the ‘subordinates’ perceptions of the extent to which supervisors engage in the sustained display of hostile verbal and nonverbal behaviors, excluding physical contact” [27] (p. 178). It is characterized by hostile verbal and nonverbal behaviors from a supervisor and has been identified as a significant workplace stressor with detrimental consequences for both employees and organizations [27,28,29,30]. One of the key outcomes of abusive supervision appears to be employee silence, which refers to the suppression of one’s voice or opinions in the workplace [31]. Silence is not simply the absence of voice [1], it also reflects a conscious decision of employees to withhold important information and suggestions about their work and organization [32]. 

There is a growing body of evidence linking abusive supervision and employee silence [4,27,31,33,34,35,36,37,38], and the theoretical argumentation on employee silence has been grounded in the COR theory [8]. This theory posits that when individuals are exposed to resource-depleting behaviors, they want to conserve their current resources while avoiding future resource loss [8]. When an employee is subjected to abusive supervision, this employee experiences a depletion of resources and thereby feels a need to conserve those resources to cope with the stressful situation. For instance, when authoritarian leaders prioritize absolute authority, displaying unfavorable responses toward those leaders could bring certain costs, such as losing social status or career promotions for the subordinates [39]. Subordinates could also hesitate to confront their abusive supervisors [40,41] and “play it safe” due to the power imbalance between supervisors and subordinates [42] (p. 221). Under such a situation, the employees might avoid speaking up, withhold their ideas, and prefer to be silent even if they believe that doing so would be in their best interests [7,37,41,42]. Those employees would, in turn, express their dissatisfaction and displeasure toward their supervisors by maintaining their silence [43]. Supporting this argument, recent research [33,44] also revealed that employees who are exposed to abusive supervision react with defensive silence to preserve their remaining resources. Thus, we propose the following:

**H1.**  *Perceptions of abusive supervision positively relate to employee silence.*

## 3. Moderating Effects: Gender Similarity and Perceived Sociodemographic Similarity

The impacts of both gender and perceived sociodemographic similarity can be explained by the premises of social identity theory. This theory [18,19] contends that individuals tend to categorize themselves and others as similar or dissimilar in different social settings concerning shared characteristics [21]. Accordingly, individuals tend to see themselves as similar or dissimilar to others concerning shared characteristics [6].

Social identity theory provides a framework that helps to explain the effects of the relational demographic factors observed in interpersonal interactions. ‘The relational demographic approach’ emphasizes “*the comparative demographic characteristics of members of dyads or groups who are in a position to engage in regular interactions*” [15] (p. 403). It analyzes the similarities and differences between people based on demographic characteristics such as age, race, gender, tenure, and educational background. The fundamental principles of this viewpoint are grounded in the similarity–attraction paradigm, which argues that individuals who share comparable attitudes or personal attributes will experience attraction toward one another [45]. 

Employees place significant importance on their interpersonal interactions with organizational authorities. According to social identity theory [15,45], in the work setting, an employee might view a supervisor who shares similarities with himself/herself as part of an in-group, and a supervisor who is different as part of an out-group. In this sense, employees may prefer interacting with individuals who are similar to them, as they perceive these interactions as validating their personal opinions and identities. Accordingly, a person’s demographic attributes, whether similar or different from those of others, would influence how those people behave with each other, which in turn affects employee outcomes. Supporting this, the literature has shown that employees tend to develop stronger ties [46] and behave more positively toward others who display a greater similarity with themselves, while they might be more aggressive toward others who display a lesser similarity [47,48].

Gender as a demographic characteristic reflects the “common membership in a salient social category” [49] (p. 356). When a subordinate and supervisor have the same gender, this similarity could serve as a signal that prompts the anticipation that both parties will hold comparable attitudes and beliefs [50]. Employees might develop stronger ties with a same-gender leader, and those strong ties may lead them to rationalize the supervisor’s behavior. On the other hand, employees who have a gender dissimilarity with their supervisor may find it difficult to form close ties, leading to a lower level of identification with the supervisor. Thus, the literature on the direct effects of gender similarity suggests that gender induces perceived similarity between leader and follower, which, in turn, leads to positive interpersonal experiences in vertical relationships [51]. 

Regarding the moderating effect of gender similarity, it is revealed that paternalistic leadership encourages silence behavior in both gender-similar and dissimilar dyadic relationships, while benevolent behaviors of leaders have a positive effect on the defensive voice of gender-similar groups [52]. Furthermore, reports indicate that subordinates with gender-similar supervisors experience a weaker indirect relationship between abusive supervision and silence via psychological distress [21]. Accordingly, employees with gender-similar supervisors are affected more strongly by their psychological state and prefer to be silent when they experience abusive leadership.

Beyond gender, the perceived sociodemographic similarities between subordinates and supervisors could also shape the relationship between abusive supervision and silence. The current study utilizes age, educational background, ethnicity, social status, and cultural similarities as sociodemographic similarities. In line with the ‘*relational demographic approach*’ and similarity–attraction paradigm, when employees and supervisors have greater perceived similarity in particular characteristics such as age [53,54], marital status [55], education [15], ethnicity [56,57], cognitive style [58,59], and power distance [53] those similarities could cultivate a stronger connection, better communication, and goal alignment, thus fostering higher levels of attraction and interaction [11] between parties. Hence, working with a similar supervisor is expected to buffer the effects of adverse conditions and could confirm the employee’s attitudes and cognitions. 

The tenets of social identity theory argue that the presence of similarity with the supervisor in terms of gender and sociodemographic characteristics has the potential to foster a sense of “we” identity among employees, leading to a sense of predictability and comfort [60], and signals an indicator of belonging to a certain group. In this regard, employees expect fair treatment and support from supervisors who share similarities [50]. However, when this expectation has been violated and when subordinates are subjected to abusive treatment from supervisors who seem similar to them, those subordinates could perceive such mistreatment as a more identity-threatening situation. In other words, the betrayal of unmet expectations could be more profound when it comes from someone ‘perceived as similar’ and could intensify the negative impact on the employee. Such a situation could increase employees’ reluctance to share their opinions and encourage them to exhibit more silent behavior. 

With the same token, gender and social background similarities are viewed as important resources at work. However, stressful situations could invalidate those resources [8]. The violation of the exchange relationship between subordinate and supervisor in terms of abusive supervision could exacerbate the feelings of resource loss on the part of subordinates who have supervisors with similar gender and sociodemographic characteristics. Thus, we propose that abusive supervision as a stressor could invalidate and/or mitigate the positive effect of those similarities on employee outcomes. Hence, employees tend to exhibit increased silence as a response to the perceived loss of resources, to prevent any additional resource depletion [21]. Based on the above arguments, we hypothesize that the more the employees perceive similarities with their supervisor, the more silence reaction they would exhibit in response to abusive supervision.

**H2.**  *Gender similarity moderates the relationship between abusive supervision and silence such that the strength of the relationship is stronger when there is a gender similarity between parties.*

**H3.**  *Perceived subordinate–supervisor sociodemographic similarity moderates the relationship between abusive supervision and silence such that the strength of the relationship is stronger when there is a perceived sociodemographic similarity between parties.*

## 4. Method

### 4.1. Participants and Procedure

This study utilized a cross-sectional design where all study variables were answered by a sample of full-time private sector employees in Türkiye. The information was gathered through an online survey. Before data collection, a participant recruitment message, which included the details of this study was prepared and posted on LinkedIn and Instagram. The recruitment message included background (i.e., the objectives of this study, name of the researchers, what participants would do, etc.) and contact information for those who want to learn more about the research and its results. The message included a link to an online survey and a QR code. The message was first sent to the people in the network of researchers; then, participants were requested to send the link to the online survey to the people in their network. Thus, the snowballing technique was utilized to recruit the participants. The individuals who participated in this research fulfilled the condition of (i) working in a private organization and (ii) having been employed under the supervision of a supervisor for a minimum of six weeks to acquire reliable evaluations of the study variables. A total of 800 people started the survey. After eliminating the incomplete responses (i.e., not responding to more than 50% of all-scale items), 742 participants remained in the data set with a response rate of 92.75%. Among those, 8 were excluded from the analysis due to excessive outlier answers. This resulted in a final sample of 734 participants (328 women, 405 men, and 1 other). The respondents worked in various industries including healthcare (44.4%), manufacturing (14.3%), hospitality and tourism (14.1%), energy (7.2%), finance (6.8%), infrastructure/energy (6%), education (4.2%), and information technologies (2.9%). The participants’ average age was 33.16 (SD = 9.3), and 48.9% of the respondents were married (SD = 7.8).

Ethical approval from the Ethical Commission Board was obtained before data collection. Participants examined the informed consent form, which contained a concise description of this study and their rights, before signing. They were provided the option to withdraw from this study. Participants were informed that their participation was entirely voluntary, ensuring the confidentiality and anonymity of their responses, and emphasizing that there were no right or wrong answers [61]. No incentives were provided for participation.

### 4.2. Measures

The research provided an online questionnaire to collect data comprising the following self-reporting scales. The questionnaire included the variables of abusive supervision, employee silence, and perceived similarity, as well as demographics (gender, gender of the supervisor, age, tenure, education, position, sector, organization type, etc.). The measures were based on existing scales, which were translated into Turkish and back-translated into English, in line with the procedure of Brislin [62].

*Abusive Supervision*. Tepper’s 15-item scale was used to measure how employees perceive abusive supervision [27]. The sample item included ‘*reminds me of my past mistakes and failures*’ and *‘puts me down in front of others*’. The respondents were requested to indicate the frequency of the supervisor’s actions using a 5-point scale (1- ‘*I cannot remember him/her ever using this behavior with me*’ to 5- ‘*he/she uses this behavior very often with me*’) in the response format. Higher scores indicated that individuals had experienced abusive supervision practices from their superiors more frequently. The Turkish translation of the instrument was also conducted [63]. The Cronbach alpha coefficient was found to be 0.92.

*Silence Behavior.* Given that it is challenging for observers to detect silence, we preferred to use self-report data to assess the silence behavior of employees similar to earlier investigations [7]. Therefore, we have used five items taken from a silence scale [64]. The responses were scored on a 5-point frequency scale, with 0 representing ‘never’ and 4 representing ‘always’ for items such as ‘*I say nothing to co-workers/others about problems I notice*’. Higher scores indicated that participants exhibited higher silence behavior. The Cronbach alpha coefficient was found to be 0.78.

*Gender Similarity*. The participants were requested to indicate the sex of their supervisor as a further demographic question. We operationalize the gender similarity variable by creating a dummy variable, with the same subordinate–supervisor gender combination coded as ‘1’ (MM: male supervisor–male subordinate and/or FF: female supervisor–female subordinate and different gender combinations coded as ‘0’ (FM: female supervisor–male subordinate and/or MF: male supervisor–female subordinate).

*Perceived subordinate–supervisor sociodemographic similarity.* We also assessed the employees’ perceptions of sociodemographic similarities to their supervisor in terms of age, educational background, ethnicity, social status, and cultural similarities. We used 5 items to measure the perceived sociodemographic similarities borrowed from the earlier studies [57,65]. The sample items were phrased as ‘*My supervisor and I are coming from similar cultural backgrounds*’, ‘*My supervisor and I share similar ethnic backgrounds*, and ‘*My supervisor and I have similar educational backgrounds*’.

All items had response alternatives ranging from strongly disagree (1) to strongly agree (5). Higher scores indicated a greater perceived similarity between subordinates and supervisors. The Cronbach alpha reliability scale was 0.74.

## 5. Results

### 5.1. Preliminary Analysis

The data were examined for entry accuracy, missing values, and adherence to normal distribution assumptions. Skewness and kurtosis values were used to assess univariate normality, whereas Mardia’s coefficient of value was employed to test multivariate normality. All skewness and kurtosis readings were below the critical threshold of ±3 [66]. No departures from multivariate normality were found.

As the data were collected from the same source, we utilized some remedies to rule out the common method variance (CMV) bias. As an ex-ante remedy, we employed a variety of scale response alternatives and anchor labels when creating the questionnaire to prevent common scale properties [67]. As an ex-post remedy, to establish discriminant validity, we employed Harman’s single-factor test and confirmatory factor analysis [68]. In particular, we conducted an Exploratory Factor Analysis with all the items of abusive supervision, silence, and perceived similarity factors. The unrotated factor analysis produced four factors, with the first factor accounting for 30.17% of the variance. This variance was below the acceptable threshold of 50% [69], indicating that no single factor accounted for a majority of the variance. Additionally, the latent common method variance factor is specified to determine whether the significance of the structural parameters changes when the latent common methods variance component is present and not present in the model [67]. Since the significance of parameters did not change, the plausible effect of common method variance was decided to be minimal, if not eliminated.

To test the validity of the factor structure of the scales and ensure whether scale items were able to measure intended constructs, we performed several confirmatory factor analyses (CFAs). In the first CFA, all the items are loaded into a one-factor solution. The model fit indices of the one-factor model suggested a poor fit to the data (*χ*^2^ (299) = 2564.45, *p* < 0.001, χ^2^/df = 8.57, GFI = 0.83, CFI = 0.724, TLI = 0.626, RMSEA = 1.10; SRMR = 0.08). In the second CFA, abusive supervision, silence, and perceived sociodemographic similarity were assumed to be three distinct factors. The model fit indices of the three-factor model yielded a good fit to the data *(χ*^2^ (296) = 980.047, *p* < 0.001, *χ*^2^*/df* = 3.31, GFI = 0.89, CFI = 0.92, TLI = 0.91, RMSEA = 0.06; SRMR = 0.05). The comparison of the three-factor model to the one-factor model resulted in a significant Chi-Square difference (Δ*x*^2^ =1584.40, *df* = 3, *p* < 0.05). This indicated the superiority of the three-factor model over the one-factor model. Overall, CFA confirms the validity of the three-factor structure and indicates that the variables represent statistically distinct constructs.

Prior to hypothesis testing, data were categorized based on the responses to the gender of the participant and the gender of each participant’s supervisor. In particular, a female participant having a female supervisor (denoted as FF) and a male participant having a male supervisor (MM) were coded under the ‘Gender Similarity’ category, whereas other combinations (i.e., the female participant having a male supervisor (FM) and male participant having female supervisor (MF) were coded under the ‘Gender Dissimilarity’ category). As seen from Table 1, gender similarity constitutes 65% of all possible gender dyads.

Table 2 displays the means, SDs, and correlations for the study variables. The hypotheses are initially supported by the relationships between the variables. As seen in Table 2, there is a positive correlation between perceptions of abusive supervision and silence behavior (*r* = 0.19; *p* < 0.01). Moreover, negative correlations were found between perceived sociodemographic similarity and perceptions of abusive supervision (*r*= −0.22; *p* < 0.01) and perceived sociodemographic similarity and silence behavior (*r*= −0.14; *p* < 0.01). The demographic variables did not significantly correlate with silence behavior; therefore, those variables were not controlled for the subsequent analyses. 

### 5.2. Results of Hypotheses Testing

We conducted two groups of regression analyses by using Model 1 of PROCESS macro [70] to test the main and interactive effects of abusive supervision perceptions and subordinate–supervisor similarities on silence behavior. With a bootstrap sample size of 5000 and a 95% confidence interval, the first group of analyses tested the main and the moderating effect of subordinate–supervisor gender similarity, and the second group of analyses tested the main and the moderating effect of perceived sociodemographic similarities (i.e., ethnicity, age, educational background, social status, and cultural) on the relationships between abusive supervision and silence behavior. Centered values of abusive supervision and perceived sociodemographic similarities were used to avoid multicollinearity problems in the analysis. The regression results for the moderation hypothesis of gender similarity are presented in Table 3.

The findings in Table 3 suggest that the main effect of abusive supervision on silence was significant (*b* = 0.21, SE = 0.04, LLCI = 0.14, ULCI = 0.29), confirming Hypothesis 1. However, neither the main effect of gender similarity (namely female subordinate having a female supervisor and male subordinate having a male supervisor) (*b* = −0.001, SE = 0.06, LLCI = −0.12, ULCI = 0.10) nor the interaction effect of gender similarity and abusive supervision (*b* = −0.01, SE = 0.09, LLCI = −0.19, ULCI = 0.16) was significant in predicting employee silence behavior. Thus, Hypothesis 2, proposing the moderation effect of gender similarity between the relationship between abusive supervision and silence was not supported.

The second group analyses tested both the main and interactive effects of abusive supervision and sociodemographic similarities between subordinates and supervisors on employee silence behavior. The sociodemographic similarity is operationalized according to the similarities based on age, ethnicity, educational background, and social status similarity. As expected, the main effects of both abusive supervision (*b* = 0.22, SE = 0.04, LLCI = 0.14, ULCI = 0.31) and perceived sociodemographic similarity (*b* = −0.12, SE = 0.04, LLCI = −0.20, ULCI = −0.04) on employee silence were found significant (see Table 4). In other words, the main effect of perceived sociodemographic similarity suggests that when the subordinates perceive more sociodemographic similarity, their silence behavior decreases. In addition to its main effect, the interaction of sociodemographic similarity with abusive supervision was also found to be significant (*b* = 0.12, SE = 0.05, LLCI = 0.03, ULCI = 0.22). Supporting Hypothesis 3, the bootstrap confidence intervals of conditional effects are significant at all levels of sociodemographic similarity (for low levels: *b* = 0.15, SH = 0.04, *t* = 3.69, *p* < 0.001, LLCI = 0.07, ULCI = 0.23; for moderate levels: *b* = 0.24, SH = 0.05, *t* = 5.22, *p* < 0.001, LLCI = 0.15, ULCI = 0.33; and for high levels: *b* = 0.31, SH = 0.06, *t* = 4.85, *p* < 0.001, LLCI = 0.18, ULCI = 0.44).

**Figure 2:**
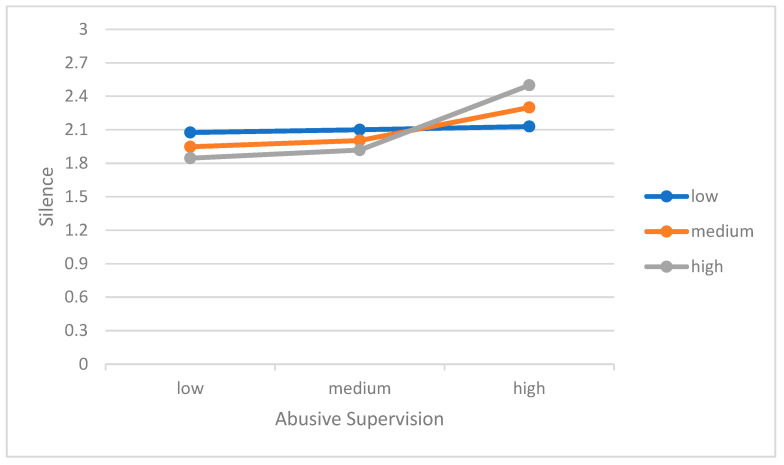
The moderating effect of sociodemographic similarity on the relationship between abusive supervision and employee silence.

To illustrate the interaction effects, we plotted the regression of the dependent variable on the outcome variable at three levels of moderating variables (1 SD below the mean, mean, and 1 SD above the mean). Figure 2 displays how the relationship between abusive supervision and employee silence behavior varies as a function of sociodemographic similarity levels. A visual inspection of the Figure suggests that the higher levels of perceived sociodemographic similarity intensify the positive relationship between abusive supervision and employee silence behavior. Thus, the form of the moderating effect of sociodemographic similarity was found to be consistent with Hypothesis 3 and suggested that the effect of abusive supervision on employee silence behavior increases when the subordinates perceive more sociodemographic similarities to their supervisors.

## 6. Discussion

Rooted in COR [71] and social identity theory [19], the current study aimed to address the main effect of abusive supervision on employee silence and the interactive effects of actual and perceived dis/similarities between subordinates and supervisors on this relationship. More specifically, this research treats gender similarity and perceived sociodemographic similarity between subordinates and supervisors as moderating variables for the abusive supervision–employee silence linkage. 

The study findings confirm Hypothesis 1, which posits the negative effect of abusive supervision on silence behavior. Consistent with the prior literature and COR theory [10,31,33], our findings show that when subordinates’ important resources are threatened and depleted by abusive supervision practices, those employees seem to adopt resource conservation strategies and distance themselves from abusive supervisors by exhibiting silence behavior [33]. Given the nature of Turkish culture [23], relatively high power distance may exacerbate the effect of abusive supervision on silence [34]. While power distance provides supervisors unlimited power and control, employees have a tendency not to question that power. Thus, the employees from high-power-distance cultures are more likely to display silence behavior as they are taught to uncritically obey orders from their supervisors [44]. 

That is to say, employees could be unwilling to share and express their constructive concerns and prefer to remain silent to protect themselves. This particular finding confirms the prior findings [16,72] which report that employees with high power distance are more susceptible to abusive supervision and exhibit more defensive and acquiescent silence behavior out of fear of recourse loss.

Regarding gender similarity, both gender similarity between subordinate and supervisor and its interaction with abusive supervision on employee silence was insignificant, rendering Hypothesis 2 unsupported. In other words, regardless of gender similarities, employees experiencing abusive supervision are more likely to remain silent at work. This finding is consistent with the suggestion that the plausible effect of gender similarity on subordinate–supervisor relationships could have been neutralized for employees perceiving abusive supervision [20]. Therefore, while gender similarity could result in better relationships between supervisors and subordinates, the presence of abusive supervision can undermine the effect of gender similarity between subordinates and superiors. The insignificant moderating effect could also be explained by the strong main effect of abusive supervision on employee silence, which could have attenuated the main and interaction effects of gender similarity. 

In contrast to the impact of gender similarity, perceived sociodemographic similarities were found to have significant main and interaction effects. Regarding the main effect, it is noteworthy that the employees are less likely to display silence behavior when they perceive sociodemographic similarities to their supervisors. However, this effect is neutralized and even altered when abusive supervision practices have been endorsed by perceived-similar supervisors. Accordingly, the positive effect of abusive supervision on employee silence behavior is found to be stronger when there is a perceived sociodemographic similarity between the employee and the supervisor. One could suggest that when multiple demographic factors intersect (age, education, ethnicity, etc.), the silence reaction can become even more complex and entrenched.

In line with the relational demography approach [15] and the prior evidence [50,51], the perceived similarity between subordinates and supervisors fosters a sense of ‘social fit’ and ‘like-mindedness’ between parties. In other words, consistent with social identity theory, high similarity between subordinates and supervisors can enhance shared thinking and establish a harmonious relationship, while fostering alignment of perspectives. Conversely, if the basic needs of the employees, such as a sense of belonging and “we” identity are threatened by the supervisor, their identification with their supervisor could be damaged [18]. Consequently, these employees who perceive sociodemographic similarities with their supervisors may view such mistreatment as a threat to their identity, leading to an increased reluctance to share their opinions and a tendency to exhibit silence behavior. In a similar vein, it is noted that employees sharing gender similarities with their supervisors are more affected by their psychological state, resulting in amplified silence in response to abusive leadership [21]. Alternatively, in collectivistic cultures, perceived similarity with the superior is argued to constitute an important condition for the establishment and preservation of a cohesive relationship [73]. In such cultural contexts, the impact of perceived sociodemographic similarities on the relationship between abusive supervision and silence could be substantial. Hence, the findings highlight the notion that employees may feel a stronger sense of betrayal when abuse comes from a supervisor they perceive as being ‘like them’, which can lead to increased hurt and withdrawal.

In light of the results, one could argue that perceived dis/similarities based on more profound factors (i.e., value congruence or social background commonalities) have a greater impact on employees’ responses to abusive supervision rather than gender dis/similarities in Turkish organizational settings. This finding converges with the finding revealing that attitudinal similarities matter more than actual similarities in explaining the quality of subordinate–supervisor relationships [74]. Moreover, it has also been contended that actual similarities, such as demographics, could affect only how people perceive others, whereas perceived similarities based on more profound factors could affect the general attitudes and behaviors of individuals at work [75]. 

## 7. Limitations and Future Research

Despite the contributions of this research to our understanding of this subject, we identified some potential limitations that should be mentioned. The first limitation is that responses came from the same source (i.e., followers), which might create common method bias. It is suggested that method bias poses less of a concern when examining moderation effects, as estimates for interaction effects remain reliable even when data are obtained from the same source [76]. Nevertheless, we adhered to several recommendations aimed at mitigating method bias, as outlined by scholars [68]. Consequently, ex-ante and ex-post analyses (Harman’s test and CFAs) indicate that common method bias was of lesser concern in our study design. The second limitation concerns the cross-sectional design of this study. Future research can utilize a longitudinal design to confirm the causal inferences. Third, this study did not measure actual abusive supervision behaviors; however, there might be discrepancies between how subordinates perceive abusive supervision and how the supervisor behaves. We intentionally chose to use self-reports because our focus was primarily on personal experiences (such as silence, perceived job insecurity, and fulfillment of needs) that are difficult for alternative sources (like colleagues or supervisors) to accurately report. Thus, future research may benefit from collecting data from multiple sources (e.g., subordinates, supervisors, actual abusive supervisor behaviors, or scales based on observers who witness mistreatment at work). Fourth, this study examines the effects of perceived similarities from the subordinate’s perspective. Future studies could focus on the opposite side of the coin by investigating the supervisors’ perspectives regarding the similarities and their outcomes.

Lastly, our data were collected in Türkiye, where national culture places high value on power distance, collectivism, and authority [23,37], so the generalizability of the results could be questioned. In a former study [37] conducted in the Turkish work context, the researchers demonstrated the adverse effects of harmful leadership practices on employee behavior. Accordingly, the suppression of one’s feelings and emotions for the sake of social harmony seems to be deeply embedded in collectivist cultures [77]. Furthermore, social identity theory argues that individuals with higher collectivism will act to benefit the group [53]. It might be possible that subordinates’ responses to abusive supervision may be more negative in Western culture compared to those in non-Western culture, and the strength of the relationships could differ concerning the moderating variables. Hence, we call for future research to evaluate our study model in a Western context and investigate the possibility of cross-cultural impacts. Last but not least, this study focused only on one outcome of employee silence behavior. We recommend exploring a wider range of employee outcomes, such as deviant workplace behaviors and unethical pro-organizational behaviors both in Western and non-Western cultures.

### Practical Implications

The current research indicates that the practice of abusive supervision could significantly influence the behaviors of employees. This study offers organizations and managers deeper insights into the reasons behind the damaging consequences abusive supervision can have on employee silence behavior. Our study results suggest that perceived sociodemographic similarity is a significant factor in predicting employee silence. Indeed, while the literature highlights the significance of perceived similarities between subordinates and supervisors on positive work outcomes, it is crucial to acknowledge that this positive effect may lead to detrimental outcomes when one is exposed to abusive supervision from supervisors perceived to be akin to oneself. Thus, the current study highlights the significance of abusive supervision on silence behavior beyond the existing knowledge. First, our study provides organizations and managers with information to develop effective intervention tools concerning abusive supervision and its consequences. Specifically, organizations may benefit from training interventions, workshops, individualized coaching, and online mentoring programs around how to effectively maintain and manage supervisors and employee behaviors. Based on our research findings, we propose that organizations adopt training interventions focused on nurturing helpful behaviors and cultivating prosocial supervision styles to enhance employees’ experiences. Leaders must recognize that they serve as representatives of their team members and thus bear a responsibility to care for their subordinates, rather than solely functioning as managerial instruments aimed at boosting productivity at the cost of their employees’ health and well-being.

Thus, through awareness and alternative intervention programs, supervisors could be more conscious of employee silence and its detrimental effects on organizations. With such awareness, organizations could develop better strategies and procedures supporting employees’ expression of their ideas and quality suggestions. Encouraging employee voice could be particularly important, especially within cultural contexts where silence is more prevalent [78]. In those cultures, employees could be reluctant to directly report such negative behaviors when they are exposed to abusive treatment from their immediate supervisors. In that case, establishing grievance mechanisms and offering anonymous feedback channels to report abusive supervision could enhance employees’ willingness to speak up. 

Organizations may benefit from considering issues of dis/similarity in their attempts to develop effective leader–follower partnerships. This study shows that subordinate–supervisor similarity does not always lead to positive outcomes and even could be harmful in unpredictable ways when it interacts with abusive and destructive leadership practices. As discussed previously, employees who perceive sociodemographic similarities with their supervisors may view such mistreatment as a threat to their identity, leading to an increased reluctance to share their opinions and a tendency to exhibit silence behavior. Therefore, including different employees with their supervisors in work groups may serve as an important intervention for preventing silence behavior. In those circumstances, ensuring that supervisors and employees are composed of varying sociodemographic attributes could also help mitigate the adverse effects of abusive supervision. 

Third, as both abusive supervision and silence could lead to stress and negative work behaviors, organizations could provide employee assistance programs [7,79] to help employees cope with both abusive supervision and silence. For instance, training programs can be specifically tailored to cultivate the abilities of subordinates to effectively manage the stress and demands imposed by their abusive supervisors [80]. Last, but not the least, organizations could encourage supervisors to communicate more frequently with their subordinates to better convey their intentions and avoid any misunderstandings [81]. 

## 8. Conclusions

We contributed to the literature by exploring how subordinates react to mistreatment from supervisors whom they perceive to be similar. Given the findings, while the current literature focuses on the positive effects of perceived subordinate–supervisor similarities, this study has important contributions, showing that how the subordinates perceive their supervisors based on the similarities has real consequences for employee silence behaviors within organizations. This study enriches our understanding of the relationship between supervisors’ abusive treatment and employee silence behavior. It is revealed that the positive effect of abusive supervision on employee silence is enhanced especially when subordinates perceive sociodemographic similarities to their supervisors. 

In this sense, policymakers could design awareness and regular training programs to encourage employees to raise concerns about abusive treatment resulting in employee silence. Besides establishing clear prohibitions and prompt reporting procedures, organizations could encourage diversity within the organization and include employees with different backgrounds to prevent silence behavior.

## Figures and Tables

**Table 1:** Gender similarity/dissimilarity: subordinate and supervisor gender combinations.

Gender Combinations	Count	Percentage (%)
FF: Female subordinate–female supervisor	159	17.7
MM: Male subordinate–male supervisor	424	47.3
FM: Female subordinate–male supervisor	233	26.0
MF: Male subordinate–female supervisor	61	6.8
Gender Similarity: FF and MM (Female subordinate–female supervisor and/ or Male subordinate–male supervisor)	583	65.1
Gender Dissimilarity: FM or MF (Female subordinate–male supervisor and/or male subordinate–female supervisor	295	32.9
Missing	18	2.0

**Table 2:** Means, standard deviations, and correlations of variables in this study.

	Mean (SD)	1	2	3	4	5	6	7
1. Abs. Sup	1.47 (0.66)	1 (0.92)						
2. Silence	2.06 (0.71)	0.19 **	1 (0.78)					
3. SD Similarity	3.31 (0.68)	−0.22 **	−0.14 **	1 (0.74)				
4. G. Similarity	-	0.01	−0.01	0.05	1			
5. Gender	-	0.05	−.04	0.00	0.47 **	1		
6. Tenure	-	0.09 *	0.01	0.03	0.03	−0.03	1	
7. Marital	-	−0.02	0.01	0.01	−0.01	−0.13	0.18 *	1

Note. The numbers in parentheses in the diagonal denote Cronbach alpha coefficients. Abs. Sup: Perceptions of Abusive Supervision, G. Similarity: Gender Similarity (0 = Gender Dissimilarity; 1 = Gender Similarity), Gender: 1 = Women, 2 = Men, * *p* < 0.05, ** *p* < 0.01.

**Table 3:** Moderation analysis for gender similarity.

Dependent Variable: Silence, F (3716) = 9.67, *p =* 0.000, R^2^ = 0.04						
Predictor	*b*	SE	*t*	*p*	LLCI	ULCI
Constant	2.06 **	0.03	78.51	0.00	2.02	2.12
Abs. Sup.	0.21 **	0.04	5.36	0.00	0.14	0.29
G. Similarity	−0.001	0.06	−0.11	0.91	−0.12	0.10
Abs.Sup * G.Similarity	−0.01	0.09	−0.15	0.88	−0.19	0.16

Notes: *b*: Unstandardized regression estimate, SE: Standard error of unstandardized estimate, LLCI: Confidence interval lower bound, UPCI: Confidence interval upper bound, Abs. Sup: Abusive Supervision; G. Similarity: Gender similarity. **: *p* < 0.01; *: *p* < 0.05.

**Table 4:** Moderation analysis for perceived sociodemographic similarity.

Dependent Variable: Silence F (3.718) = 14.40, *p =* 0.000, R^2^ = 0.06						
Predictor	*b*	SE	*t*	*p*	LLCI	ULCI
Constant	2.08	0.03	78.82	0.00	2.02	2.13
Abs. Sup	0.22	0.04	5.19	0.00	0.14	0.31
SD Similarity	−0.12 **	0.04	−3.08	0.00	−0.20	−0.04
Abs. Sup * SD Similarity	0.12 *	0.05	2.62	0.01	0.03	0.22
Conditional effects of abusive supervision at values of the moderator (SD Similarity):						
SD Similarity	Effect	SE	*t*	*p*	LLCI	ULCI
−0.60 (−1 SD)	0.15	0.04	3.69	0.00	0.07	0.23
0.12 (mean)	0.24	0.05	5.22	0.00	0.15	0.33
0.70 (+1 SD)	0.31	0.07	4.84	0.00	0.18	0.44

Notes: *b*: Unstandardized regression estimate, SE: Standard error of unstandardized estimate, LLCI: Confidence interval lower bound, UPCI: Confidence interval upper bound, Abs. Sup: Abusive supervision; SD: Similarity: Sociodemographic similarity. **: *p* < 0.01; *: *p* < 0.05.

## Data Availability

The data for this study are available upon request.
